# Absence of mutations in four genes encoding for congenital cataract and expressed in the human brain in Tunisian families with cataract and mental retardation

**DOI:** 10.1186/1471-2415-11-35

**Published:** 2011-11-21

**Authors:** Manèl Chograni, Myriam Chaabouni, Faouzi Mâazoul, Hedi Bouzid, Abdelhafid Kraiem, Habiba B Bouhamed Chaabouni

**Affiliations:** 1 University Tunis Elmanar, Faculté de Médecine de Tunis, Laboratoire Génétique Humaine, Tunis, Tunisia; 2 Congenital and Hereditary Disorders Department, Charles Nicolle hospital, Tunis, Tunisia; 3 Department of Neonatology, Military hospital, Tunis, Tunisia; 4 Opthalmology Department, Habib Thameur hospital, Tunis, Tunisia

## Abstract

**Background:**

To identify the genetic defect associated with autosomal recessive congenital cataract (ARCC), mental retardation (MR) and ARCC, MR and microcephaly present in most patients in four Tunisian consanguineous families.

**Methods:**

We screened four genes implicated in congenital cataract by direct sequencing in two groups of patients; those affected by ARCC associated to MR and those who presented also microcephaly. Among its three genes *PAX6*, *PITX3 *and *HSF4 *are expressed in human brain and one gene *LIM2 *encodes for the protein MP20 that interact with the protein galectin-3 expressed in human brain and plays a crucial role in its development. All genes were screened by direct sequencing in two groups of patients; those affected by ARCC associated to MR and those who presented also microcephaly.

**Results:**

We report no mutation in the four genes of congenital cataract and its flanking regions. Only variations that did not segregate with the studied phenotypes (ARCC associated to MR, ARCC associated with MR and microcephaly) are reported. We detected three intronic variations in *PAX6 *gene: IVS4 -274insG (intron 4), IVS12 -174G>A (intron12) in the four studied families and IVS4 -195G>A (intron 4) in two families. Two substitutions polymorphisms in *PITX3 *gene: c.439 C>T (exon 3) and c.930 C>A (exon4) in one family. One intronic variation in *HSF4 *gene: IVS7 +93C>T (intron 7) identified in one family. And three intronic substitutions in *LIM2 *gene identified in all four studied families: IVS2 -24A>G (intron 2), IVS4 +32C>T (intron 4) and c.*15A>C (3'-downstream sequence).

**Conclusion:**

Although the role of the four studied genes: *PAX6*, *PITX3*, *HSF4 *and *LIM2 *in both ocular and central nervous system development, we report the absence of mutations in all studied genes in four families with phenotypes associating cataract, MR and microcephaly.

## Background

Congenital cataracts show considerable clinical and locus heterogeneity and represent one of the major causes of vision loss world-wide [1]. Cataracts can be isolated or can occur in association with a large number of different metabolic diseases or genetic syndromes [2]. Non syndromic congenital cataract has an estimated frequency of 1-6 per 100,000 live births [3].

More than 20 genes have been implicated in human congenital cataract, most of them are responsible for the autosomal dominant trait than the autosomal recessive one [4,5] in these can be divided into two clusters according to the stage of lens development at which they are involved. The first group contains genes that determine lens structure and genes related to structure, including *crystallins*, *Bfsp*, *connexions *and *MIP *[4-6].

Among genes encoding for membrane proteins (MIP), *LIM2 *gene (lens intrinsic membrane protein-2) involved in autosomal recessive congenital cataract [7,8]. It encodes an abundant integral lens membrane protein MP20 which is a new regulatory protein important for mammalian lens fiber cell junctional formation [9]. Lens-specific MP20 is classified as a member of the PMP22/EMP/MP20 subfamily of tetraspanins [10] and adds to a growing list of ligands of galectin-3[11], a known adhesion modulator that is expressed by microglial cells of adult brain [12] and contributes to injury in the deep gray matter areas of the brain [13].

The second group of genes including transcription factors *PAX6*, *PITX3 *and *HSF4 *member of the heat shock transcription factor (HSF) family [6]. These three transcription factors are expressed in the human brain and led to autosomal dominant or autosomal recessive congenital cataract or both.

*PAX6 *gene (Paired box homeotic gene 6), 11p13, a paired box transcription factor, was described the among the wide set of genes responsible for autosomal dominant congenital cataract (ADCC) [14] that is expressed in the developing eye, brain, spinal cord and pancreas and seems to play a crucial role in the developing of the central nervous system by transcriptional regulation of various target genes [15,16].

*PITX3 *gene (Pituitary homeobox gene 3) mapped on 10q24.32, is also responsible for ADCC and expressed in the developing lens, skeletal muscle, and dopaminergic neurons of the substantia nigra in the brain [17].

*HSF4 *gene (Heat-shock transcription factor 4) localized on 16q21-q22.1 and for which mutations are associated with both autosomal dominant [18] and autosomal recessive congenital cataract (ARCC) [19-21]. In the human, *HSF4 *is widely expressed, especially in the heart, brain, skeletal muscle, lung and pancreas [22,23].

Based on these observations, we analysed the four genes: *LIM2*, *PAX6*, *PITX3 *and *HSF4 *in four consanguineous unrelated Tunisian families with ARCC associated with mental retardation (MR) for two patients belonging to the same family and ARCC associated to MR and microcephaly for the others from the four studied families.

## Methods

### Subjects and sample collection

**Figure 1:**
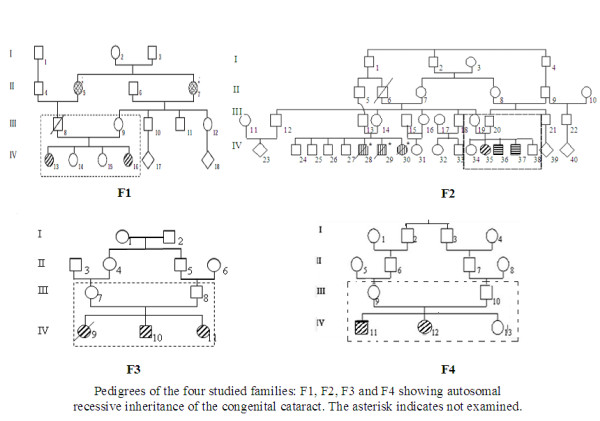
**Pedigrees of the four studied families**. Filled squares with oblique lines pointing to the right describe patients with Congenital cataract, Mental retardation and Microcephaly, filled squares with horizontal lines represent patients with Congenital cataract and Mental retardation, filled squares with vertical lines describe patients with Mental retardation and filled circles represent patient with Late-onset cataract. The asterisk indicates not examined patients.

We evaluated seventeen patients (7 parents, 10 patients including 9 affected and 1 normal from family F1) belonging to four Tunisian unrelated families (Figure 1) recruited from Congenital and Hereditary Disorders Department at Charles-Nicolle Hospital (Tunis, Tunisia).

All four families were of Tunisian origin and were enrolled in a genetic research program in the laboratory of Human Genetics, in the faculty of medicine (Tunis, Tunisia) because of two affected brothers belonging to family F2 with ARCC and MR and seven affected patients from the four studied families (F1, F2, F3, F4) with ARCC, MR and microcephaly.

The nine patients (5 males, 4 females) were born from healthy and consanguineous parents. Pedigrees' patterns are concordant with autosomal recessive inheritance for the four families (Figure 1). Their mean age was 23 years, ranging from 8 to 41 years. We noted that the father from family F1 was dead after au traumatic accident. Cataracts were reportedly present since birth in all patients. None had glaucoma before or after the extraction of cataracts.

The cataracts were of the posterior polar type and bilateral in all patients except of the affected child IV11 belonging to family F4. All patients had undergone cataract extraction early in life. Visual acuity was preserved in all patients except of the affected child IV12 from family F4 who showed decreased visual acuity and alteration of the pigment epithelium. We denoted the presence of retinal dystrophy and strabismus in patient IV16 belonging to family F1. We underlined also the presence of nystagmus, microphthalmia and strabismus in patient IV11 from family F3 whose brother IV10 presented also microphthalmia.

Significant physical disability became apparent for all patients by the age of 15 to 18 months when they failed to walk. They also had a significant delay in speech development. In fact, the nine affected patients were developmentally delayed with mild to moderate mental retardation with no dysmorphic features. Microcephaly was present in all of them except the two brothers IV36 and IV37 belonging to family F2. Additional features are shown in table 1.

**Table 1:** Clinical features of the 9 affected patients belonging to the four studied families: F1, F2, F3 and F4.

*Familles*	F1		F2			F3		F4	
***Patients***	**IV13**	**IV16**	**IV35**	**IV36**	**IV37**	**IV10**	**IV11**	**IV11**	**IV12**

**Weigt (Kg)**	N	N	N	N	N	N	N	N	N

**Height (cm)**	N	N	N	N	N	N	N	N	N

**Cataract**	unilateral	bilateral	bilateral	bilateral	bilateral	bilateral	bilateral	unilateral	bilateral

**Age of cataract**	3 years	3 years	2 years	3 years	2 years	6 months	1 year	3 years	3 years

**Microcephaly**	-5.5 DS	-6.7 DS	-2.8 DS	N	N	-3.5 DS	-3 DS	-5.6 DS	-3.8 DS

**MR**	moderate	moderate	moderate	moderate	mild	moderate	moderate	moderate	moderate

**Age of MR**	3 years	3 years	2 years	3 years	2 years	1 year	3 years	3 years	3 years

**Developmental** **delay**	-Could not learn nor write. -Troubles of eloculation -Running all alone.		-Walked at age of 2 years. -Difficult to shake hands and close eyes.	-Walked at 2 years. -Spoke at 5 years. -Absence of anatomy.	Difficult to learn.	-Spoke at 2 years. -Speech with monosyllabic words.		Walked and spoke at ages of 2 years.	
								
						IV10 -Sit with supports since 5 months. IV 10 -Walk with assistance gained.			

**MRI**	N	Small parietal ischemic lesion	N	N	N	-Anomaly if Dandy-Walker. -Thinning of corpus callosum.		N	N

**Others**	Slight axial hypotonia	-Retinal dystrophy -Strabismus	-	. -	-	-Microphtalmia. -Axial hypotonia. -Intrauterine growth retardation. IV10- Bilateral cryptorchidism operated.		-	-Alteration of the pigment epithelium. -Leucorea -Decreased visual acuity.

MR: Mental Retardation, MRI: Magnetic Resonance Image, N: normal, -: no other features

Age of MR = age of the patient at the first time referred to our department.

Magnetic resonance imaging (MRI) of the brain was normal in all screened patients except for the presence of a small ischemic parietal lesion in patient IV16 from family F1 and the presence of Dandy-Walker anomaly with thinning of corpus callosum in patients IV10 and IV11, belonging to family F3, whose sister IV9 showing MR, bilateral congenital cataract, microcephaly and epilepsy was dead at 3 years.

Biological investigations (karyotyping with R-banding) revealed normal karyotypes; 46,XX for females, 46,XY for males (600 bands resolution) and normal metabolic screening including Fehling reaction and thin layer chromatography of reducing sugars, plasmatic amino acid and urine organic acid chromatography for all patients.

Genomic DNA of affected and unaffected members (10 siblings, 7 parents) was extracted from peripheral blood leukocytes by the standard proteinase-K extraction consisting on: lysis of red blood cells by RBC (Red Blood Cells) Lysis Buffer (155 mM NH4Cl, 10 mM KHCO3, 0.5 EDTA, pH 7.5) and white blood cells by a WBC (White Blood Cells) Lysis Buffer (1 mM Na-EDTA, 5 mM Tris HCl pH 7.5), treatment of the lysate with a mixture of detergent composed of SDS (Sodium Dodecyl Sulfate or sacrosyl and proteinase K) in order to liberate the DNA and digest the associated proteins, precipitation of the DNA in the form of filaments by absolute ethanol and finally diluation of the DNA in T10E1 Buffer (Tris 10 mM, EDTA 0.1-1 mM), and stored in 10 ml Vacuum tube sterile containing 100 μl of 0.1 M EDTA.K3.

## Patients and parents for minors gave informed consent

In this study, the researches carried out on human are in compliance with the Helsinki Declaration and ethics committee Charles Nicolle hospital, Tunis has given approval for this study.

### DNA amplification and sequencing

All exons and intron-exon junctions of the four genes: *LIM2 *(4 exons), *PAX6 *(14 exons), *PITX3 *(4exons), *HSF4 *(13 exons) were amplified from genomic DNA by polymerase chain reaction (PCR) using primers chosen by Primer3 (Primer3-web/hldocs/inpat-040.htm).

The genes sequences were retrieved from the Ensembl database; (ENST00000221973 for *LIM2*/ENST00000419022 for *PAX6*/ENST00000370002 for *PITX3 *and ENST00000264009 for *HSF4*).

The amplification reactions were performed by using 100 ng of each patients' genomic DNA as a template, 20 pmol of each primer (Biomatik, Canada), a MgCl_2 _concentration depending upon the exon amplified, 1.5 Units of Taq DNA polymerase recombinant (Invitrogen, Carlsbad), and 1.25 mM dNTP (Promega, Madison) in a total volume of 50 μl. PCR consisted on 35 cycles and was carried out in an automated thermal cycle GeneAmp PCR System 9700 (Applied Biosystems, Foster city) under the following conditions: 95°C for 5 min, 95°C for 30 s, then 52-60°C for 30 s, and elongation at 72°C for 30 s, followed by one cycle of extension at 72°C for 7 min.

The amplified products were purified (Wizard^® ^SV Gel and PCR Clean-Up System Kit; Promega) and sequenced (Big Dye Terminator Cycle Sequencing Ready Reaction; DNA Sequencing Kit; ABI PRISM 3130) in the forward and reverse directions. Sequencing results were visualized and data were computer analyzed using Sequencing Analysis 5.2 and SeqScape softwares (Applied Biosystems, Foster City).

## Results

Screening of nine affected siblings from four Tunisian families for *LIM2*, *PAX6*, *PITX3 *and *HSF4 *pathogenic mutations revealed no mutation in the corresponding exons of each gene.

For *LIM2 *gene (exon 1 is an UTR for non-translated region), sequence analysis showed novel intronic changes involving modifications: IVS2 -24A>G in intron 2; IVS4 +32C>T in intron 4; c.*15A>C in 3' down-stream sequence. All affected and unaffected individuals were homozygous for the modified alleles.

The same for *PAX6 *gene, in that we detected novel intronic variations entailing: IVS4 -274insG in intron 4, IVS12 -174G>A in intron 11, identified in all four families and IVS4 -195G>A in intron 4 identified only in families F3 and F4 (we considered the isoform *PAX6 *(+5a) for mutation nomenclature).

For *PITX3 *gene, we identified two substitutions polymorphisms in family F**1: **an already reported silent polymorphism, c.439C>T single nucleotide polymorphism (SNP rs2281983), in exon 3 that conserved the same amino acid Isoleucine (I) in the protein sequence p. I95I. Another unreported variation c.930C>A in exon 4, resulting in a proline to leucine substitution at position 258 of the protein. These two exonic changes are not included in a conserved domain of *PITX3 *(we considered the first nucleotide of the cDNA sequence nucleotide number one).

And for *HSF4 *gene, we revealed one novel intronic variation; IVS7 +93C>T in intron 7 detected only in family F1.

The variations identified in these four screened genes did not segregate with the phenotypes (ARCC, MR and ARCC, MR and microcephaly) because of the absence of correlation between genotypes (homozygous/heterozygous) and autosomal recessive inheritance of such studied phenotypes in the four pedigrees (Table 2).

**Table 2:** Polymorphic changes identified in *PAX6*, *PITX3 *and *HSF4 *genes in the studied patients from the four Tunisian families (F1, F2, F3, and F4)

*Familles*	*Patients*	*PAX6 intronic variations*			*PITX3 subtitution polymorphisms*		*HSF4 intronic variation*	
	
		IVS4 -274insG	IVS12 -174G>A	IVS4 -195G>A	c.439 C>T (exon 3)	c.930 C>A (exon 4)	IVS7 +93C>T	
**F1**	III9	homozygous	heterozygous	wild type	heterozygous	heterozygous	heterozygous	
	
	IV13	homozygous	homozygous	wild type	heterozygous	heterozygous	heterozygous	
	
	IV14	homozygous	homozygous	wild type	wild type	heterozygous	wild type	
	
	IV16	homozygous	homozygous	wild type	wild type	heterozygous	wild type	

**F2**	III19	homozygous	heterozygous	wild type	wild type	wild type	wild type	
	
	III20	homozygous	homozygous	wild type	wild type	wild type	wild type	
	
	IV35	homozygous	homozygous	wild type	wild type	wild type	wild type	
	
	IV_36_	homozygous	homozygous	wild type	wild type	wild type	wild type	
	
	IV37	homozygous	homozygous	wild type	wild type	wild type	wild type	

**F3**	III9	homozygous	heterozygous	heterozygous	wild type	wild type	wild type	
	
	III10	homozygous	heterozygous	heterozygous	wild type	wild type	wild type	
	
	IV11	homozygous	heterozygous	wild type	wild type	wild type	wild type	
	
	IV12	homozygous	heterozygous	heterozygous	wild type	wild type	wild type	

**F4**	III7	homozygous	heterozygous	heterozygous	wild type	wild type	wild type	
	
	III8	homozygous	heterozygous	heterozygous	wild type	wild type	wild type	
	
	IV10	homozygous	wild type	homozygous	wild type	wild type	wild type	
	
	IV11	homozygous	heterozygous	heterozygous	wild type	wild type	wild type	

For each novel variation identified in the four studied genes, 50 normal unrelated controls were screened and results are indicated in table 3.

**Table 3:** Frequency of the novel SNPs, identified in the four screened genes, on 50 normal controls.

*Gene/Variation*		*Homozygous (for the variation)*	*Heterozygous (for the variation)*	*Wild type*
	IVS2 -24A>G	11 (22%)	0	39 (78%)
	
***LIM2***	IVS4 +32C>T	39 (78%)	11 (22%)	0
	
	c.*15A>C	35 (70%)	15 (30%)	0

	IVS4 -274insG	46 (92%)	4 (8%)	0
	
***PAX6***	IVS12 -174G>A	0	9 (18%)	41 (82%)
	
	IVS4 -195G>A	11 (22%)	25 (50%)	14 (28%)

***PITX3***	c.930C>A (exon 4)	0	0	50 (100%)

***HSF4***	IVS7 +93C>T	0	0	50 (100%)

Concerning *LIM2 *and *PAX6 *genes, control samples were either heterozygous or homozygous for the variable alleles which confirmed the fact that these variations did not segregate with the studied phenotype in the four families.

But for *PITX3 *and *HSF4 *genes although variations were absent in 50 ethnically matched control samples, there is no segregation between these detected polymorphisms and the studied phenotype (association between congenital cataract, mental retardation and microcephaly) in family F1.

## Discussion

Congenital cataracts are common major abnormalities of the eye, which frequently cause blindness in infants [24]. It may occur as an isolated anomaly, as part of generalized ocular development defects, or as a component of a multisystem disorder [24]. In fact, association of cataract with congenital anomalies, mental retardation and microcephaly is reported in several cases with chromosomal anomalies and syndromes from genic origins [25-27].

Until today no candidate gene has been reported responsible for such phenotypes: association between congenital cataract, MR and congenital cataract, MR and microcephaly, so we tried to focus on genes already described in ADCC and/or ARCC and expressed in the human brain (*PAX6*, *PITX3 *and *HSF4*). And we chose to investigate the role of *LIM2 *gene in such phenotypes regarding the interaction between the proteins MP20 and galectin-3 in lens fiber cell.

MP20, a member of the tetraspanin superfamily, is the most abundant integral membrane protein of lens fiber cells, which appears to be distributed uniformly in the plasma membrane but also occurs in distinct membrane junctional domains early during embryonal development [9,28-30].Mutations in MP20 severely disrupt normal crystalline fiber cell arrangement in the lens and cause cataractogenesis [7,8,31]. Tetraspanins form contacts with other cells or the extracellular matrix by binding to other tetraspanins, to adhesion receptors such as integrins, and to extracellular proteins [32-34]; in this context, recently MP20 and galectin-3 were identified to co-localize in selected areas of the cell plasma membrane and biochemical analysis confirmed that MP20 and galectin-3 interact with each other [11]. In fact, galectin-3 is a multifunctional protein, which occurs early during embryonal development, with reported involvement in development, oncogenesis, and inflammation [35]. It was found to be expressed by microglial cells [12], detected in a number of astrocytes in adult rat brain [36] and leads to injury in the deep gray matter areas of the brain [13]. In addition, *LIM2 *has been implicated in autosomal recessive cataract and reported for the first time on 2002 by Pras and co-workers [7]. By sequencing of *LIM2*, they revealed a Phe105Val mutation leading to a phenotype with a late-onset of cataract characterised by nuclear opacities, mild to moderate visual loss in three affected sibs whose healthy brother showed mental retardation with clear lens. On 2008, a second mutation Gly154Glu was reported by Ponnam and co-workers [8] in a phenotype involving congenital cataracts with severe visual impairment indicated by the presence of nystagmus and amblyopia but also with no sign of MR.

For *PAX6 *gene, it was first described as a candidate for human aniridia and ADCC but not for ARCC [37]. PAX6 expression is not restricted to the eye and appears to be crucial for brain development [38]. In order to elucidate this hypothesis, Dansault et al. [39] reported 14 affected members carrying a p.S74G mutation in exon 6 of *PAX6 *gene. All of them were suffering from diverse congenital ocular abnormalities including congenital cataracts, diverse neurological manifestations and variable cognitive impairments. Recently, Chien et al. [40] had identified a p.R317X PAX6 mutation in a patient (familial case) suffering from cataract, aniridia, nystagmus and was developmentally delayed. So these two reports proved that the *PAX6 *gene has a key role as a master regulator in the development of the eye and central nervous system.

As for *PITX3*, it was demonstrated to cause cataract and anterior segment mesenchymal dysgenesis (ASMD) in several families from different ethnic origins [41,42]. It has been described only in ADCC but not in ARCC [43]. Recently, Bidinost and coworkers (2006) reported one large Lebanese family where patients with a heterozygous mutation in *PITX3 *(650delG) had posterior polar cataracts (PPCs), while patients with the same mutation but with the homozygous state had a more severe ocular effect with severe microphthalmia associated with developmental delay and mental retardation. In addition, the two homozygous brothers are offspring of a consanguineous marriage, and their parents, who presented with PPCs, were examined through the 28 affected members and were heterozygous for the reported deletion. The neurologic phenotype of the homozygous patients implies an essential role for *PITX3 *in normal ocular and central nervous system development and this was the first report in which a potential new role of *PITX3 *in the development of the nervous system has been proved.

The last studied gene is *HSF4 *which was identified sufficiently important to lens development [44,45] and disruption of the *HSF4 *gene leads to both autosomal dominant and ARCC (11-13). Bu et al. [18] reported, on 2002, four different missense mutations, within the *HSF4 *DNA binding domain, in patients with autosomal dominant lamellar and Marner cataracts from a large Chinese family. On 2004, Smaoui et al. [19] reported for the first time a *HSF4 *homozygous splice mutation in intron 12 (c.1327+4A>G) causing the skipping of exon 12 and leading to the installation of ARCC in a consanguineous Tunisian family. Then, Sajjad and coworkers [20] identified a novel *HSF4 *gene mutation (p.R405X) causing ARCC in a large consanguineous family from Pakistan. In the human, HSF4 is widely expressed, especially in the brain, heart, skeletal muscle, lung and pancreas [22,23].

Taking these results further, we analysed *LIM2*, *PAX6*, *PITX3 *and *HSF4 *genes in four consanguineous Tunisian families with nine affected patients showing ARCC, MR for two brothers from F2 and ARCC, MR and microcephaly for the seven other patients belonging to the four families, but we did not identify any pathogenic mutation.

Only novel intronic variations; IVS2 -24A>G, IVS4 +32C>T, c.*15A>C detected in *LIM2 *gene, IVS4 -274insG, IVS12 -174G>A, IVS4 -195G>A identified in *PAX6 *gene and IVS7 +93C>T in *HSF4 *gene. And substitution polymorphisms; a reported one c.439 C>T (rs2281983) and a novel SNP c.930 C>A revealed respectively in exons 3 and 4 of *PITX3 *gene. These modifications did not segregate with studied phenotypes (ARCC, MR and ARCC, MR and microcephaly).

These findings did not exclude the role of *LIM2*, *PAX6*, *PITX3 *and *HSF4 *gene in both ocular and central nervous system but it underlined the fact that these transcription factors (*PAX6*, *PITX3*), heat-shock transcription factor (*HSF4*) and lens intrinsic membrane protein (*LIM2*) could not be responsible for the association between ARCC, MR and ARCC, MR and microcephaly in the four studied Tunisian families in spite of their expression in the human brain (*PAX6*, *PITX3*, *HSF4*) or their interaction with proteins expressed in human brain (*LIM2*).

## Conclusion

In conclusion, a genome wide scan must be performed for these four families in order to identify candidate regions so that candidate gene(s) leading to such association.

## List of abbreviations

We use ARCC for autosomal recessive congenital cataract, MR for mental retardation, *PAX6 *for Paired box homeotic gene 6, *PITX3 *for Pituitary homeobox gene 3, *HSF4 *for Heat-shock transcription factor 4, *LIM2 *for lens intrinsic membrane protein-2 and ADCC for autosomal dominant congenital cataract.

## Competing interests

The authors declare that they have no competing interests.

## Authors' contributions

**MC **carried out thechoice of genes, the molecular genetic study, the sequence alignement and drafted the manuscript, **MyC **participated in the choice of the gene, **FM **carried out the examination of the patients, **HB**: participated in the clinical study of the patients, **AK**: carried out the ophthalmologic examination of the other patients and **HBC **conceived of the study, and participated in its design and coordination and helped to draft the manuscript.

All authors read and approved the final manuscript.

## Pre-publication history

The pre-publication history for this paper can be accessed here:

http://www.biomedcentral.com/1471-2415/11/35/prepub
